# Supplementary material for: MetaRibo-Seq measures translation in microbiomes
Source: Nat Commun. 2020 Jun 29;11:3268. doi: 10.1038/s41467-020-17081-z (PMC7324362; doi:10.1038/s41467-020-17081-z)
Supplement: Supplementary file 10 — Supplementary Data 7 [file 41467_2020_17081_MOESM10_ESM.zip › File2/Confidence_High_Taxonomy/119236_out.krona.html]

Javascript must be enabled to view this page.

members
magnitude
magnitudeUnassigned
count
unassigned
taxon
rank

119236\_out

3

2759
2
superkingdom

kingdom
2
4751

subkingdom
451864
2

2
4890
phylum

subphylum
147538
2

class
147550
2

2
222544
subclass

order
2
5114

2
767018
family

2
238244
genus


SRS051031\_contig\_number\_36581SRS1041145\_contig\_number\_30894
species
2
238245


SRS076929\_contig\_number\_contig-100\_1856.122931
1
